# CD19 chimeric antigen receptor T‐cell efficacy and toxicity in adults with Richter's transformation and response to bridging therapy

**DOI:** 10.1111/bjh.20227

**Published:** 2025-07-13

**Authors:** Angela Hwang, Amy A. Kirkwood, Maeve O'Reilly, Lorna Neill, Caroline Besley, Shankara Paneesha, Mathew Amrith, Paul Maciocia, Andrea Kuhnl, David Irvine, Jane Norman, Ahmed Abdulgawad, Carlos Gonzalez Arias, Sunil Iyengar, Helen Marr, Satyen Gohil, Claire Roddie

**Affiliations:** ^1^ Department of Haematology University College London Hospitals London UK; ^2^ UCL Cancer Institute, University College London London UK; ^3^ Cancer Research UK and UCL Cancer Trial Centre UCL Cancer Institute, University College London London UK; ^4^ Department of Haematology University Hospitals Bristol and Weston Bristol UK; ^5^ Department of Haematology Queen Elizabeth Hospital Birmingham UK; ^6^ Department of Haematology King's College Hospital London UK; ^7^ Department of Haematology Queen Elizabeth University Hospital Glasgow UK; ^8^ Department of Haematology Manchester Royal Infirmary Manchester UK; ^9^ Department of Haematology The Christie Hospital Manchester UK; ^10^ Department of Haematology Royal Marsden Hospital London UK; ^11^ Department of Haematology Freeman Hospital Newcastle upon Tyne UK

**Keywords:** CAR‐T, DLBCL, Richter's transformation

To the Editor,

Despite novel agents, Richter's transformation (RT) still carries a dismal prognosis with a median overall survival (OS) of 6–12 months.^1^, ^2^ While CD19‐targeting chimeric antigen receptor (CAR)‐T therapy (CD19CAR‐T) has transformed outcomes in relapsed/refractory (r/r) large B‐cell lymphoma (LBCL), CAR‐T efficacy in RT is incompletely understood as RT patients were excluded from pivotal trials.^3^, ^4^

However, two emerging real‐world datasets of CD19CAR‐T for r/r RT demonstrate impressive overall response rates (ORR) and median OS rates of 63% and 8.5 months (*n* = 63 patients),^5^ and 57% and 9.9 months (*n* = 30 patients)^6^ respectively. In the latter analysis, only 14/30 patients received commercial CAR‐T products and experienced poorer median progression‐free survival (PFS) and OS (2.9 and 4.3 months) compared to the total cohort, which included patients receiving experimental CAR‐T products (6.7 and 9.9 months).^6^

Separately, a DESCAR‐T registry study of CD19CAR‐T for RT demonstrated slightly lower ORR and complete response (CR) rates (50%; 42%) with higher toxicity compared to *de novo* LBCL.^7^

In the United Kingdom (UK), CD19CAR‐T was approved for r/r RT in December 2018. Here, we evaluate the feasibility, safety and efficacy of commercially available CD19CAR‐T for r/r RT in the UK, highlighting clinical features associated with PFS and OS.

This retrospective intention‐to‐treat (ITT) analysis included r/r RT patients approved for third‐line axicabtagene ciloleucel (axi‐cel) or tisagenlecleucel (tisa‐cel) across nine sites by the UK National CAR‐T Clinical Panel (NCCP) from 2018 to 2023. Further details on methods and statistics are found in Supporting Information.

Regarding patient and disease characteristics, 27 patients were approved for CD19CAR‐T therapy and all underwent leukapheresis (Table 1; Figure S1). Median age at CAR‐T approval was 63 years (range: 25–78). Of 27 patients, 21 (78%) had a preceding diagnosis of chronic lymphocytic leukaemia (CLL) with a median interval of 4.4 years to RT diagnosis (interquartile range [IQR]: 2.0–6.1; range: 0.7–11.7).

**TABLE 1:** Baseline characteristics and bridging therapy.

	All patients	Infused
	*N* = 27	*N* = 23
CAR‐T product, *N* (%)		
Axi‐cel	23 (85.2)	20 (87.0)
Tisa‐cel	4 (14.8)	3 (13.0)
Age at NCCP		
Median (range)	63 (25–78)	62 (25–78)
Sex, *N* (%)		
Male	14 (51.9)	11 (47.8)
Female	13 (48.1)	12 (52.2)
LDH grouped, *N* (%)		
Normal	7 (35.0)	7 (35.0)
>Upper limit of normal	10 (50.0)	10 (50.0)
>2× upper limit of normal	3 (15.0)	3 (15.0)
Missing/unknown	7	3
Lymphocyte count at apheresis		
Median (IQR), range	1.6 (0.9–2.6), 0.4–6.8	1.6 (0.9–2.6), 0.4–6.8
Haematotox score (group), *N* (%)		
Low (0–1)	11 (47.8)	11 (47.8)
High 2+	12 (52.2)	12 (52.2)
Missing/unknown	0	0
CLL		
Cytogenetics at diagnosis, *N* (%)		
Normal	4 (30.8)	3 (25.0)
Abnormal	9 (69.2)	9 (75.0)
del(11q)	2 (15.4)	2 (16.7)
del(11q) and del(13q)	1 (7.7)	1 (8.3)
del(13q) and ATM partial	1 (7.7)	1 (8.3)
del(17p)	2 (15.4)	2 (16.7)
Other	3 (23.1)	3 (25.0)
Unknown	14	11
*del*(*17p*) or *TP53*‐mutated? *N* (%)		
No	14 (63.6)	11 (61.1)
Yes	8 (36.4)	7 (38.9)
Missing/unknown	5	5
*IGHV‐*unmutated? *N* (%)		
No	2 (20)	1 (12.5)
Yes	8 (80)	7 (87.5)
Missing/unknown	17	15
Prior lines of therapy (CLL)		
Median (range)	1 (0–5)	1 (0–5)
Richter's transformation		
Cell of origin, *N* (%)		
GCB^a^	1 (4.0)	1 (4.5)
Non‐GCB	24 (96.0)	21 (95.5)
Missing/unknown	2	1
CD5, *N* (%)		
Positive	7 (100.0)	7 (100.0)
Missing/unknown	20	16
Clonal relationship, *N* (%)		
Related	3 (75.0)	3 (75.0)
Tested, unknown	1 (25.0)	1 (25.0)
Not tested/unknown	23	19
Prior lines of therapy		
Median (range)	2 (2–5)	2 (2–5)
Prior lines of therapy (CLL + RT)		
Median (range)	4.0 (2–8)	3 (0–4)
Prior treatment with both BTKi and BCL2i (CLL or RT), *N* (%)		
No	10 (37.0)	9 (39.1)
Yes	17 (63.0)	14 (60.9)
Bridging therapy		
Bridging type, *N* (%)		
Systemic	22 (81.5)	18 (78.3)
Radiotherapy	2 (7.4)	2 (8.7)
CMT	3 (11.1)	3 (13.0)
Bridging regimen, *N* (%) [systemic/CMT only]		
RBP	21 (84.0)	17 (81.0)
R‐pola	2 (8.0)	2 (9.5)
Pirtobrutinib	1 (4.0)	1 (4.8)
Cyclo‐dex	1 (4.0)	1 (4.8)
Response to bridging, *N* (%)		
CMR	2 (7.4)	2 (8.7)
PR	9 (33.3)	9 (39.1)
SD	1 (3.7)	1 (4.3)
PD	15 (55.6)	11 (47.8)

Abbreviations: BCL2i, B‐cell lymphoma 2 inhibitor; BTKi, Bruton's tyrosine kinase inhibitor; CAR‐T, chimeric antigen receptor T‐cell; CLL, chronic lymphocytic leukaemia; del, deletion; CMR, complete metabolic response; CMT, chemotherapy; cyclo‐dex, cyclophosphamide + dexamethasone; GCB, germinal centre B‐cell; IQR, interquartile range; LDH, lactate dehydrogenase; NCCP, National CAR T‐cell Clinical Panel; PD, progressive disease; PR, partial response; RBP, rituximab + bendamustine + polatuzumab vedotin; R‐pola, rituximab + polatuzumab vedotin; RT, Richter's transformation; SD, stable disease.

^a^ The GCB patient was confirmed CD5 positive.

Of 27 patients, 25 had histology demonstrating non‐germinal centre LBCL and/or CD5 positivity. Central histology reports were not available for two patients (Table 1). Six patients (22%) had a concomitant diagnosis of CLL at time of RT diagnosis. Where available, cytogenetic and molecular status for CLL/RT revealed 17p deletion (del(17p)) or a TP53 mutation in 8/22 patients (36%) and unmutated immunoglobulin heavy chain variable region (IGHV) gene status in 8/10 patients (80%). Clonality assessment is not routinely available or performed in the UK and results were only available in three patients.

Patients received a median of one prior line of therapy for CLL (range: 0–5) including Bruton's tyrosine kinase inhibitor (BTKi) therapy in 10/23 patients (43%), B‐cell lymphoma 2 inhibitor (BCL2i) therapy in 9/23 patients (39%) and phosphoinositide 3‐kinase inhibitor (PI3ki) therapy in 2/23 patients (7%) (Table 1; Table S1). Patients received a median of two prior lines of therapy (range: 2–5) for RT, including allogeneic stem cell transplantation in two patients.

At leukapheresis, the median peripheral blood lymphocyte count was 1.57 × 10^9^/L (range: 0.4–6.8). Of 27 CAR‐T products, 26 (96%) met release criteria. One product failed on cell viability criteria by 2%.

All 27 patients received bridging therapy (BT): 23/27 (85%) received polatuzumab‐based chemo‐immunotherapy (CIT); 2/27 (7%) received non‐polatuzumab CIT; 2/27 (7%) received radiotherapy and 1/27 (4%) received BTKi, which was ceased prior to lymphodepletion (Table 1).

Response assessment post‐BT revealed complete response (CR) in 2/27 patients (7%), partial response (PR) in 9/27 (33%), stable disease (SD) in 1/27 (4%) and progressive disease (PD) in 15/27 (56%). Four patients (15%) did not proceed to CAR‐T due to rapid PD post‐BT (all received rituximab + bendamustine + polatuzumab vedotin [RBP]) and all died within 3 months of approval.

Post‐BT lactate dehydrogenase (LDH) was elevated in 13/20 patients (65%) and baseline haematotoxicity (HT) score was ‘high risk’ in 12/23 (52.2%).

Of 27 patients, 23 (85%) received CAR‐T infusion, of whom 20/23 (87%) received axi‐cel and 3/23 (13%) received tisa‐cel, with a median vein to vein time of 38 days (range: 27–87). In contrast to other reports, patients did not receive concurrent BTKi therapy.^5^

Regarding toxicity, any grade cytokine release syndrome (CRS) or immune effector cell‐associated neurotoxicity syndrome (ICANS) affected 21/23 (91%) and 8/23 (35%) patients respectively, with ≥Grade 3 CRS and ICANS in 3/23 (13%) and 1/23 (4%) patients respectively, primarily in patients with PD post‐BT and elevated LDH pre‐lymphodepletion (median: 365; range: 204–1124).

At month 1 (M1) post‐CAR‐T infusion, ≥Grade 3 neutropenia and thrombocytopenia affected 12/22 (54.5%) and 8/22 (36.3%) patients respectively. This resolved in all but two cases by month 3 (M3). Higher HT score was associated with ≥Grade 3 thrombocytopenia at M1 (high vs. low risk, 4/4 vs. 0/9; *p* = 0.029). Infections affected 10/22 (45.5%) patients and are detailed in the Supporting Information: ‘Results’ section and outlined in Table 2.

**TABLE 2:** Toxicity.

Toxicity	All
	*N* = 23
	*N* (%)
CRS	
0	2 (8.7)
1	5 (21.7)
2	13 (56.5)
3	2 (8.7)
5	1 (4.4)
**3–5**	**13.0% (95% CI: 2.8–33.6)**
ICANS	
0	15 (65.2)
1	4 (17.4)
2	3 (13.0)
3	1 (4.3)
**3–5**	**4.3% (1.1–21.9)**
Grade 3–4 neutropenia 1 month^a^	
No	8 (47.1)
Yes	9 (52.9)
Grade 3–4 thrombocytopenia 1 month^a^	
No	13 (76.5)
Yes	4 (23.5)
Grade 3–4 neutropenia 3 months^a^	
No	11 (91.6)
Yes	1 (8.3)
Grade 3–4 thrombocytopenia 3 months^a^	
No	11 (91.6)
Yes	1 (8.3)
Post CAR‐T infection	
No	12 (54.6%)
Yes	10 (45.5%)
COVID‐19	6
Bacterial LRTI	1
Pneumocystis pneumonia	1
Invasive fungal lung	1
Viral URTI	2
CMV retinitis	1
*Escherichia coli* bacteraemia	1
Rotavirus	1
*Yersinia enterocolitica*	1
Other bacterial infection: *Staphylococcus haemolyticus*, *Staphylococcus hominis*, *Enterococcus faecalis + Escherichia coli* UTI, *Staphylococcus epidermidis*, *Corynebacterium*	1
Missing	1

Abbreviations: CAR‐T, chimeric antigen receptor T cell; CI, confidence interval; CMV, cytomegalovirus; CRS, cytokine release syndrome; ICANS, immune effector cell‐associated neurotoxicity syndrome; LRTI, lower respiratory tract infection; URTI, upper respiratory tract infection; UTI, urinary tract infection.

^a^ For 1‐ and 3‐month cytopenia; patients with progressive disease (PD) at 1 or 3 months have been excluded. No haemophagocytic lymphohistiocytosis (HLH) reported.

For 1‐ and 3‐month cytopenia; patients with progressive disease (PD) at 1 or 3 months have been excluded. No haemophagocytic lymphohistiocytosis (HLH) reported.

Regarding response rates and survival, in the ITT cohort (*N* = 27), the 2‐year OS from CAR‐T approval was 42.6% (95% confidence interval [CI]: 21.6–62.2).

For infused patients, ORR at M1 was 69.6%, with CR in 12/23 (52%), PR in 4/23 (17%), SD in 1/23 (4%) and PD in 5/23 (22%). By M3, three CR and PR patients developed PD and two PR patients converted to CR (Table S2).

With a median follow‐up of 22.3 months (IQR: 12.7–23.5), the median PFS and OS from infusion were 14.3 months (IQR: 1.3–not reached [NR]) and 19.4 months (IQR: 2.5–NR).

At 2 years, PFS was 45.7% (95% CI: 24.2–64.9; Figure 1A) and OS was 49.7% (95% CI: 25.0–70.3; Figure 1B) with 11/23 (48%) patients still in CR at last follow‐up (Figure 1C).

**FIGURE 1:**
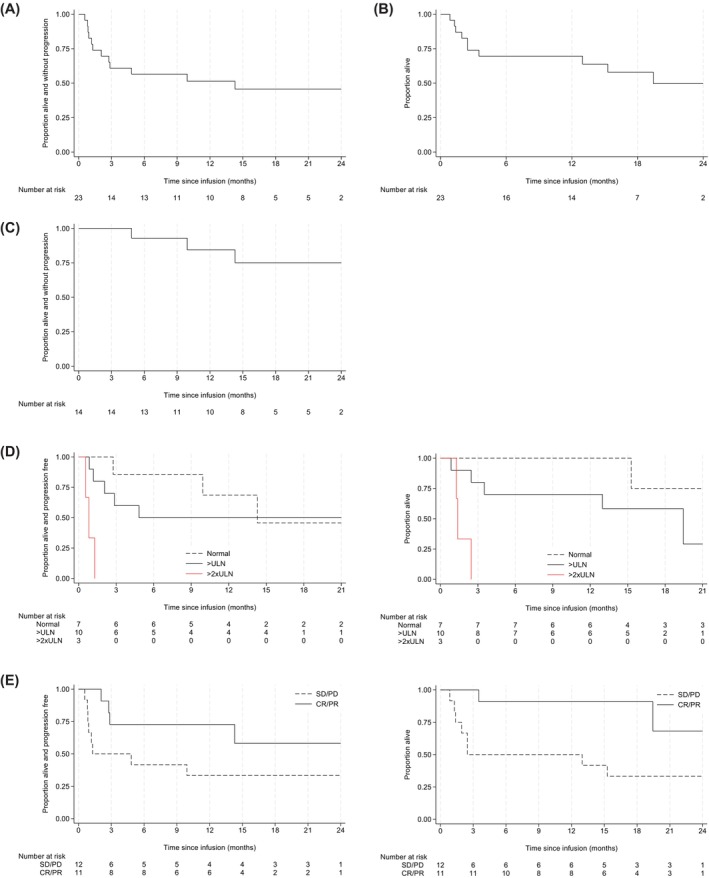
(A) Overall progression‐free survival (PFS). Median PFS from infusion: 14.3 months (IQR: 1.3–NR); 2‐year PFS: 45.7% (95% CI: 24.2–64.9). (B) Overall survival (OS). Median OS from infusion: 19.4 months (IQR: 2.5–NR); 2‐year OS: 49.7% (95% CI: 25.0–70.3). (C) PFS in patients with complete response post CAR‐T; 2‐year PFS: 75.0% (95% CI: 40.3–91.3). (D) Outcomes by response to bridging, PFS (HR responder vs. non responder: 0.38 [95% CI: 0.11–1.27], *p* = 0.12); and OS (HR responder vs. non responder: 0.20 [95% CI: 0.04–0.97], *p* = 0.045). (E) Outcomes by lactate dehydrogenase (LDH). PFS (HR [as continuous, for an increase of 1× ULN]: HR 2.42 [1.42–4.12], *p* = 0.001); OS (HR [as continuous, for an increase of 1× ULN]: 4.41 [95% CI: 0.51–38.2]). CAR‐T, chimeric antigen receptor T cell; CI, confidence interval; CR, complete response; HR, hazard ratio; IQR, interquartile range; NR, not reached; PD, progressive disease; PR, partial response; SD, stable disease; ULN, upper limit of normal.

Factors associated with PFS and OS in univariable analysis include LDH, response to BT and number of prior therapeutic lines (Table S3).

Consistent with previous reports, high LDH was associated with inferior PFS (hazard ratio [HR] = 2.08, 95% CI: 1.30–3.33; *p* = 0.002) and OS (HR = 2.47, 95% CI: 1.39–4.40; *p* = 0.002) (Figure 1D).

CR or PR response to BT was associated with superior OS (HR = 0.2, 95% CI: 0.04–0.97; *p* = 0.045) and a similar but non‐significant association with PFS (HR = 0.38, 95% CI: 0.11–1.27; *p* = 0.12) (Figure 1E).

More treatment lines for RT (HR = 1.99 for each additional line, 95% CI: 1.09–363; *p* = 0.025), prior BTKi (HR = 4.62, 95% CI: 1.23–17.31; *p* = 0.023) and prior BCL2i (HR = 4.51, 95% CI: 1.33–15.28; *p* = 0.023) were all associated with inferior PFS.

del(17p) and TP53 mutation were associated with significantly worse PFS (HR = 4.17, 95% CI: 1.11–15.64; *p* = 0.034) but not OS (HR = 3.62, 95% CI: 0.94–13.98; *p* = 0.062). Only one known IGHV‐unmutated patient was infused with CAR‐T, so impact on PFS and OS could not be assessed. Currently 1/7 TP53‐mutated and 1/8 IGHV‐unmutated patients are alive and in remission at 12.7 and 22.3 months of follow‐up respectively.

Including non‐responders, 11/23 (48%) patients relapsed at a median of 2.1 months post CAR‐T (IQR: 0.9–7.4; range: 0.6–14.3): 9/11 with LBCL, 1/11 with CLL/LBCL and 1/11 unknown. Seven (64%) patients received salvage therapy (Table S4).

Ten (43%) patients died at a median of 5.4 months (IQR: 1.3–16.6) post CAR‐T due to PD in 8/10, grade 5 CRS in 1/10 and pneumonia in 1/10.

In summary, chemo‐refractory RT carries a dismal prognosis, but CAR‐T therapy holds substantial promise.

Here, we demonstrate that CAR‐T for RT is feasible. Despite CLL/RT T‐cell ‘fitness’ concerns, CAR‐T manufacture was successful in 26/27 (96%) patients. Furthermore, 23/27 (85%) leukapheresed patients were infused, which is superior to the UK LBCL CAR‐T experience.^8^

Real‐world data suggest a relatively high incidence of high‐grade CRS and ICANS in r/r RT. Kittai et al. reported ≥G3 CRS and ICANS in 16% and 37% of patients, respectively,^5^ and a DESCAR‐T analysis reported ≥G3 ICANS in 25%.^7^ In our cohort, high‐grade immunotoxicity was relatively infrequent and G3–4 events predominantly arose in patients with SD/PD responses to BT. Furthermore, grade 3‐4 cytopenias beyond M3 were rare (2/23, 8.6%) and the incidence of infection was not dissimilar to CAR‐T in LBCL.^8^, ^9^

While CAR‐T response rates in CLL are historically lower than for LBCL,^10^, ^11^ early data suggest this may not be true for RT.^5^, ^6^, ^12^ In our analysis, ORR at M1 was 69%, consistent with other real‐world datasets^5^, ^6^ and with LBCL.^8^

However, in contrast to the short duration CAR‐T responses described by Kittai et al.^5^ and Benjamini et al.,^6^ we observed durable responses (2‐year PFS and OS, 45.7% and 49.7%; higher than the upper CIs in the Kittai study). While our study populations were similar with respect to age, prior therapeutic lines and LDH, our UK cohort included fewer patients with prior BTKi or BCL2i exposure: 61% (Table 1) versus 84% (Kittai) and 89% (Benjamini). Our study also had fewer high molecular risk patients, that is, 8/22 (36%) had a del(17p) and/or TP53 mutation and 8/10 (80%) IGHV‐unmutated versus 20/40 (50%) and 52/60 (86.7%), respectively, in the Kittai cohort^5^ and 5/12 (42%) and 4/7 (57%), respectively, in the Benjamini paper,^6^ albeit incomplete molecular datasets were reported in all.

In our analysis, inferior PFS and OS were associated with more prior lines of treatment and higher LDH. Thus, CAR‐T for RT may be more effective if used earlier in the disease course and in the setting of lower burden disease.

While BT to reduce disease burden is not well characterised in RT, our experience in LBCL suggests that a CR or PR response to BT is most likely with polatuzumab‐based BT and associated with superior PFS and OS.^13^ In contrast to the heterogeneous BT approaches described in other real‐world RT CAR‐T datasets,^5^, ^6^ here, 85% of patients received polatuzumab‐based BT and 48% achieved CR or PR post‐BT. It is possible that more effective BT options may contribute to improved CAR‐T outcomes for r/r RT.

Post CAR‐T relapse conferred a dismal outcome despite salvage therapy (median OS: 5.4 months). For those at highest risk of relapse (more prior lines, higher LDH or high‐risk molecular phenotypes) combinatorial CAR‐T and BTKi therapy or pre‐emptive consolidation with allogeneic stem cell transplantation may improve outcomes and requires prospective evaluation in clinical studies.

This retrospective analysis is limited by small patient numbers which precluded multivariate analysis meaning we are unable to exclude the possibility confounding effects in our risk factor analyses. As with other datasets, we did not have complete molecular disease profiling meaning the true risk of this group and comparability to others are hard to assess. Nevertheless, this study demonstrates that judicious patient selection and effective BT can lead to PFS and OS outcomes in RT similar to those with LBCL with manageable toxicity. Looking forward, CAR‐T for r/r RT in second line requires evaluation and may begin to address and overcome the high‐risk profile for relapse that we have observed with extensive pretreatment for RT.

## AUTHOR CONTRIBUTIONS

AH, AAK, SG and CR designed the project. AH, MOR, LN, CB, SP, MA, AK, DI, JN, AA, SI and HM compiled the clinical data. AAK analysed the data. AH, AAK and CR wrote the manuscript. All authors edited and reviewed the manuscript.

## CONFLICT OF INTEREST STATEMENT

AH has received honorarium from Kite/Gilead. AAK has received honoraria from Kite/Gilead, Janssen MOR has served on advisory boards and received honoraria from Kite/Gilead, Novartis and Janssen. CB has received honoraria and/or served on advisory boards for Kite, Novartis, Takeda and Janssen. He has undertaken consultancy and holds patents with Novalgen and UCL Business. PM holds patents with UCL Business, holds equity in Autolus and has received research funding from Autolus. AK has received honoraria from Kite/Gilead, Roche, BMS, Abbvie and travel grants from Kite/Gilead and Astra Zeneca. DI has received honoraria from Kite/Gilead. JN has received honorarium from Kite/Gilead. AA has received honoraria from Kite/Gilead. CGA has served on advisory boards and received honoraria from Kite/Gilead and Novartis; received research funding from Kite/Gilead; and conference sponsorship from Kite/Gilead and Novartis. SI has received honoraria and/or served on advisory boards for Astra Zeneca, Beigene, Kite/Gilead, MSD and Takeda. SG has received honoraria and/or served on advisory boards for Abbvie, AstraZeneca, Beigene, Electra Therapeutics, Gilead, EUSA/Recordati, Takeda and Janssen. He has undertaken consultancy and holds patents with Freeline, Novalgen and UCL Business. CR has served on advisory boards and/or received honoraria from Kite/Gilead, Novartis, Autolus, Johnson&Johnson, Bristol Myers Squibb, Cellistic and Kyverna. The remaining authors have nothing to disclose.

## ETHICS APPROVAL STATEMENT

Ethical approval for this project was obtained (REC reference: 24/EM/0221, IRAS project ID: 336254).

## PATIENT CONSENT STATEMENT

Patients provided informed consent for the collection of minimally identifiable data in line with EBMT policy.

## Supporting information


Data S1.

## Data Availability

De‐identified data that support the findings of this study are available on request from the corresponding author.
