# Supplementary material for: CD19 chimeric antigen receptor T‐cell efficacy and toxicity in adults with Richter's transformation and response to bridging therapy
Source: Br J Haematol. 2025 Jul 13;207(2):648–53. doi: 10.1111/bjh.20227 (PMC12378962; doi:10.1111/bjh.20227)

**TITLE:**

‘CD19 CAR T-cell efficacy and toxicity in adults with Richter’s Transformation and response to bridging therapy’

**SUPPLEMENTARY METHODS:**

In the UK, CD19CAR-T was approved by the National Institute for Health and Care Excellence (NICE) for r/r RT in December 2018. Data cut-off was 1st April 2024. Choice of CAR T-cell product was as at the discretion of the treating centre. Central histopathology review verified RT diagnosis. No patients had central nervous system involvement as this was an exclusion criterion.

Lymphodepletion (LD) comprised fludarabine and cyclophosphamide. CAR-T product selection was at the discretion of the treating centre. Cytokine release syndrome (CRS) and immune effector cell-associated neurotoxicity syndrome (ICANS) grade was assessed by ASTCT criteria^1^, haematological toxicity as by CTCAE criteria version 5.0, and haematotoxicity (HT) by the immune-effector-cell-associated haematotoxicity score (ICAHT)^2,3^.

Positron emission tomography-computerised tomography (PET-CT) response as per the Lugano Criteria was assessed at months 1, 3, and 6 post CAR-T^4^.

Progression free survival (PFS) and overall survival (OS) were measured from date of infusion (or approval for ITT population) until date of first event. Patients without an event were censored at date last seen. Kaplan-Meier analysis using Cox regression/log-rank test comparisons were done for PFS and OS. Differences in discrete variables were compared using Fisher’s exact tests/Chi-squared tests for trend, and continuous variables using Wilcoxon Mann-Whitney tests.

**SUPPLEMENTARY RESULTS:**

***Patient and Disease Characteristics***

26/27 (96%) CAR-T products met release criteria**.** 1/27 products failed on cell viability criteria by 2%, but the product was approved by the UK out-of-specification (OOS) products committee and the patient was infused.

***Toxicity***

Infections affected 10/22 (45.5%) patients including COVID-19 in 6/22 (27.3%), bacteraemia in 1/22 (4.5%), and isolated cases of pneumonia due to pneumocystis jirovecii, fungal or viral organisms, cytomegalovirus retinitis, and rotavirus diarrhoea (Table 2).

***Response rates and survival***

Prior BTKi or BCL2i were also associated with increased total number of RT and CLL treatment lines (median 5 vs 2.5;p=0.001 and 5 vs 3;p=0.0026) which was also associated with inferior PFS and OS.

**REFERENCES:**

1. Lee DW, Santomasso BD, Locke FL, Ghobadi A, Turtle CJ, Brudno JN, et al. ASTCT Consensus Grading for Cytokine Release Syndrome and Neurologic Toxicity Associated with Immune Effector Cells. Biol Blood Marrow Transplant. 2019 Apr 1;25(4):625–38.

2. National Cancer Institute. Common Terminology Criteria for Adverse Events (CTCAE). 2017; Available from: https://ctep.cancer.gov/protocoldevelopment/electronic_applications/docs/CTCAE_v5_Quick_Reference_5x7.pdf

3. Rejeski K, Subklewe M, Aljurf M, Bachy E, Balduzzi A, Barba P, et al. Immune effector cell–associated hematotoxicity: EHA/EBMT consensus grading and best practice recommendations. Blood. 2023 Sep 7;142(10):865–77.

4. Cheson BD, Fisher RI, Barrington SF, Cavalli F, Schwartz LH, Zucca E, et al. Recommendations for Initial Evaluation, Staging, and Response Assessment of Hodgkin and Non-Hodgkin Lymphoma: The Lugano Classification. J Clin Oncol. 2014 Sep 20;32(27):3059–67.

**SUPPLEMENTARY TABLE 1**: Prior lines of therapy for CLL and RT

|  |  | **All patients** | **Infused** | **Not infused** |
| --- | --- | --- | --- | --- |
|  |  | **N=27** | **N=23** | **N=4** |
| **Prior CLL treatment** | |  |  |  |
| **Choice of first line CLL therapy, N (%)** | |  |  |  |
|  | Acalabrutinib | 1 (5.9) | 1 (7.7) | 0 |
|  | Alemtuzumab + methylprednisolone | 1 (5.9) | 1 (7.7) | 0 |
|  | Fludarabine + cyclophosphamide | 1 (5.9) | 1 (7.7) | 0 |
|  | FCMR | 1 (5.9) | 1 (7.7) | 0 |
|  | FCR | 6 (35.3) | 4 (30.8) | 2 (50.0) |
|  | Ibrutinib | 2 (11.8) | 1 (7.7) | 1 (25.0) |
|  | Obinutuzumab+Chlorambucil | 1 (5.9) | 0 | 1 (25.0) |
|  | Obinutuzumab+Venetoclax | 1 (5.9) | 1 (7.7) | 0 |
|  | R-CHOP | 1 (5.9) | 1 (7.7) | 0 |
|  | Radiotherapy | 1 (5.9) | 1 (7.7) | 0 |
|  | Surgical excision + radiotherapy | 1 (5.9) | 1 (7.7) | 0 |
|  | No treatment | 10 | 10 | 0 |
| **CLL treatment given (any line)** | |  |  |  |
|  | Ibrutinib, N (%) | 9 (33.3) | 8 (34.8) | 1 (25.0) |
|  | Chemotherapy, N (%) | 11 (40.7) | 8 (34.8) | 3 (75.0) |
|  | Rituximab, N (%) | 10 (37.0) | 7 (30.4) | 3 (75.0) |
|  | Idelalisib, N (%) | 2 (7.4) | 2 (8.7) | 0 |
|  | Venetoclax, N (%) | 10 (37.0) | 9 (39.1) | 1 (25.0) |
|  | Acalabrutinib, N (%) | 3 (11.1) | 2 (8.7) | 1 (25.0) |
|  | Obinutuzumab, N (%) | 3 (11.1) | 2 (8.7) | 1 (25.0) |
|  | Radiotherapy, N (%) | 2 (7.4) | 2 (8.7) | 0 |
|  | Allogeneic transplant, N (%) | 1 (3.7) | 1 (4.3) | 0 |
| **Prior Richter’s therapy** | |  |  |  |
| **First line RT therapy, N (%)** | |  |  |  |
|  | R-CHOP | 25 (92.6) | 21 (91.3) | 4 (100.0) |
|  | R-ESHAP | 1 (3.7) | 1 (4.3) | 0 |
|  | Pirtobrutinib | 1 (3.7) | 1 (4.3) | 0 |
| **Second line RT therapy, N (%)** | |  |  |  |
|  | Acalabrutinib | 2 (7.4) | 2 (8.7) | 0 |
|  | Mosunetuzumab | 2 (7.4) | 2 (8.7) | 0 |
|  | R-GCVP | 1 (3.7) | 1 (4.3) | 0 |
|  | R-CHOEP | 1 (3.7) | 1 (4.3) | 0 |
|  | R-CHOP | 1 (3.7) | 1 (4.3) | 0 |
|  | R-DHAP | 1 (3.7) | 1 (4.3) | 0 |
|  | R-GDP | 13 (48.1) | 11 (47.8) | 2 (50.0) |
|  | R-GemOx | 3 (11.1) | 2 (8.7) | 1 (25.0) |
|  | R-ICE | 3 (11.1) | 2 (8.7) | 1 (25.0) |
| **Third line RT therapy, N (%)** | |  |  |  |
|  | R-GDP | 3 (30.0) | 3 (30.0) | 0 |
|  | R-GemOx | 1 (10.0) | 1 (10.0) | 0 |
|  | R-IVE | 2 (20.0) | 2 (20.0) | 0 |
|  | Pirtobrutinib | 2 (20.0) | 2 (20.0) | 0 |
|  | Rituximab-bendamustine | 1 (10.0) | 1 (10.0) | 0 |
|  | Mini-BEAM | 1 (10.0) | 1 (10.0) | 0 |
|  | No third line | 17 | 13 | 4 |
| **Fourth line therapy, N(%)** | |  |  |  |
|  | RBP | 1 (33.3) | 1 (33.3) | 0 |
|  | Gemcitabine monotherapy | 1 (33.3) | 1 (33.3) | 0 |
|  | Pirtobrutinib | 1 (33.3) | 1 (33.3) | 0 |
|  | No fourth line | 24 | 20 | 4 |
| **Fifth line therapy, N(%)** | |  |  |  |
|  | Radiotherapy | 1 (50.0) | 1 (50.0) | 0 |
|  | Cyclophosphamide-dexamethasone | 1 (50.0) | 1 (50.0) | 0 |
|  | No fifth line | 25 | 21 | 4 |
| **Previous allogeneic transplant for RT** | |  |  |  |
|  | No | 25 (92.6) | 21 (91.3) | 4 (100.0) |
|  | Yes | 2 (7.4) | 2 (8.7) | 0 |

**Abbrevations:** FCMR, fludarabine + cyclophosphamide + mitoxantrone + rituximab; FCR, fludarabine + cyclophosphamide + rituximab; R-CHOP, rituximab + cyclophosphamide + doxorubicin + vincristine + prednisolone; R-ESHAP, rituximab + etoposide + methylprednisolone + cytarabine + cisplatin; R-GCVP, rituximab + gemcitabine + cyclophosphamide + vincristine + prednisolone; R-CHOEP, rituximab + cyclophosphamide + doxorubicin + etoposide + prednisolone; R-DHAP, rituximab + dexamethasone + cytarabine + cisplatin; R-GDP, rituximab + gemcitabine + dexamethasone + cisplatin; R-GemOx, rituximab + gemcitabine + oxaliplatin; R-ICE, rituximab + ifosfamide + carboplatin + etoposide; R-IVE, rituximab + ifosfamide + etoposide + epirubicin; BEAM, carmustine + etoposide + cytarabine + melphalan; RBP, rituximab + bendamustine + polatuzumab vedotin

**SUPPLEMENTARY TABLE 2**: Responses at M1, M3 and M6

| **Time point/response** | | **N=23** |
| --- | --- | --- |
|  |  | **N (%)** |
|  |  |  |
| **Month 1** | |  |
|  | CR | 12 (52.2) |
|  | PR | 4 (17.4) |
|  | SD | 1 (4.4) |
|  | PD | 5 (21.7) |
|  | Died | 1 (4.4) |
|  | **ORR** | **16 (69.6)** |
| **Month 3** | |  |
|  | CR^1^ | 13 (56.5) |
|  | PD | 3 (13.0) |
|  | PD pre M3 | 3 (13.0) |
|  | Died | 4 (17.4) |
|  | **ORR** | **16 (69.5)** |
| **Month 6** | |  |
|  | CR^1^ | 13 (56.5) |
|  | PD pre M6 | 6 (26.1) |
|  | Died | 4 (17.4) |
|  | **ORR** | **13 (56.5)** |
|  |  |  |

**Abbreviations:** CR, complete response; PR, partial response; SD, stable disease; PD, progressive disease. ^1^Includes 1 patient who had consolidation RT after Month 1 (PR)

**SUPPLEMENTARY TABLE 3**: Univariate analysis, Risk factors for PFS and OS

|  |  | **PFS** | | | **OS** | | |
| --- | --- | --- | --- | --- | --- | --- | --- |
|  |  | **Events/N** | **HR (95% CI)** | **p-value** | **Events/N** | **HR (95% CI)** | **p-value** |
|  |  |  |  |  |  |  |  |
| **Number of lines for RT**  **(increase of one line)** | | 12/23 | 1.99 (1.09 – 3.63) | 0.025 | 10/23 | 1.78 (0.96 – 3.31) | 0.069 |
|  | |  |  |  |  |  |  |
| **Total lines: CLL + RT**  **(increase of one line)** | | 12/23 | 1.55 (1.15 – 2.08) | 0.004 | 10/23 | 1.45 (1.04 – 2.04) | 0.030 |
|  | |  |  |  |  |  |  |
| **Bridging response** | |  |  |  |  |  |  |
|  | No | 8/12 | 1.00 | 0.12 | 8/12 | 1.00 |  |
|  | Yes | 4/11 | 0.38 (0.11 – 1.27) |  | 2/11 | 0.20 (0.04 – 0.97) | 0.045 |
|  |  |  |  |  |  |  |  |
| **LDH (increase of 1 ULN)** | | 11/20 | 2.08 (1.30 – 3.33) | 0.002 | 9/20 | 2.47 (1.39 – 4.40) | 0.002 |
|  |  |  |  |  |  |  |  |
| ***TP53* mutated** | |  |  |  |  |  |  |
|  | No | 5/11 | 1.00 | 0.034 | 4/11 | 1.00 | 0.062 |
|  | Yes | 4/7 | 4.17 (1.11 – 15.64) |  | 5/11 | 3.62 (0.94 – 13.98) |  |
| ***IGHV* unmutated** | |  |  |  |  |  |  |
|  | No | 0/1 | - | 0.21 | 0/1 | - | 0.38 |
|  | Yes | 6/7 | - |  | 6/7 | - |  |
|  |  |  |  |  |  |  |  |
| **Previous BTKi** | |  |  |  |  |  |  |
|  | No | 3/12 | 1.00 | 0.023 | 3/s14 | 1.00 | 0.13 |
|  | Yes | 9/11 | 4.62 (1.23 – 17.31) |  | 7/9 | 2.86 (0.73 – 11.11) |  |
|  |  |  |  |  |  |  |  |
| **Previous BCL2i** | |  |  |  |  |  |  |
|  | No | 4/14 | 1.00 | 0.016 | 4/14 | 1.00 | 0.096 |
|  | Yes | 8/9 | 4.51 (1.33 – 15.28) |  | 6/9 | 2.94 (0.83 – 10.48) |  |
| **Mosunetuzumab** | |  |  |  |  |  |  |
|  | No | 10/21 | 1.00 | 0.059 | 8/21 | 1.00 | 0.021 |
|  | Yes | 2/2 | 5.17 (0.94 – 28.48) |  | 2/2 | 7.56 (1.36 – 41.87) |  |
|  |  |  |  |  |  |  |  |
| **Response to first line therapy for RT** | |  |  |  |  |  |  |
|  | No | 7/12 | 1.00 | 0.44 | 7/12 | 1.00 | 0.30 |
|  | Yes | 5/10 | 0.63 (0.20 – 2.02) |  | 3/10 | 0.48 (0.12 – 1.89) |  |
|  |  |  |  |  |  |  |  |

**Abbreviations:** LDH, lactate dehydrogenase; BTKi, Bruton Kinase inhibitor; BCL2i, BCL2 inhibitor

**SUPPLEMENTARY TABLE 4:** Choice of salvage therapy post CAR-T relapse or progression

| **Further treatment/response** | | **N(%)** |
| --- | --- | --- |
|  |  |  |
| **Ibrutinib** | | 1 (14.3) |
|  | *PD* | *1* |
| **Pirtobrutinib** | | 1 (14.3) |
|  | *PR* | *1* |
| **Pirtobrutinib + radiotherapy** | | 1 (14.3) |
|  | *CR* | *1* |
| **RBP** | | 2 (28.6) |
|  | *CR* | *1* |
|  | *PR* | *1* |
| **Radiotherapy** | | 1 (14.3) |
|  | *PD* | *1* |
| **Radiotherapy, pirtobrutinib, glofitamab** | | 1 (14.3) |
|  | *PR* | *1* |
|  |  |  |

**Abbreviations:** PD, progressive disease; PR, partial response; CR, complete response; RBP, rituximab + bendamustine + prednisolone

**SUPPLEMENTARY FIGURE 1**: Patient flow chart


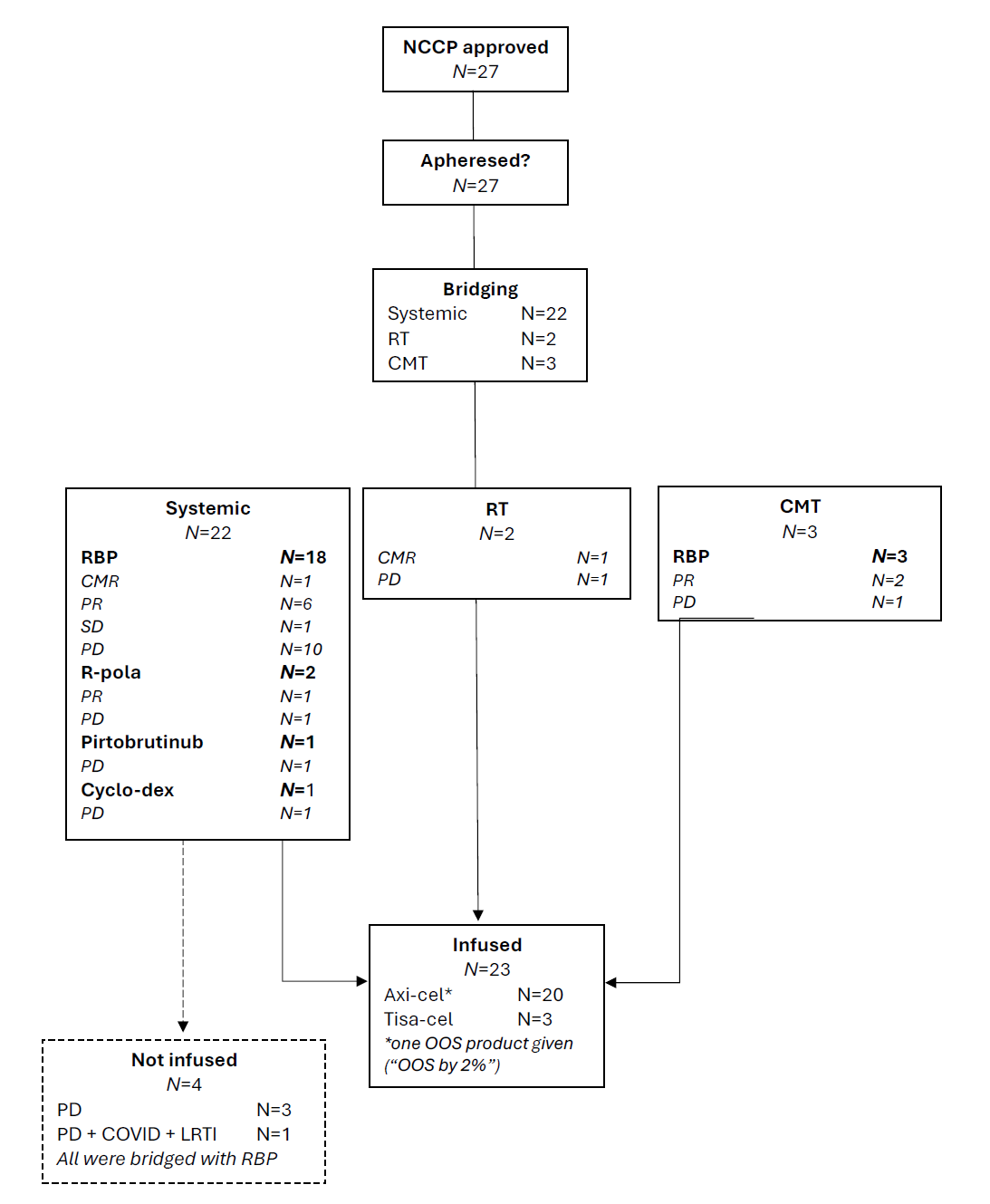

Supplement: Supplementary file 1 — Data S1. [file BJH-207-648-s001.docx]
